# How to Be a Male at Different Elevations: Ecology of Intra-Sexual Segregation in the Trawling Bat *Myotis daubentonii*

**DOI:** 10.1371/journal.pone.0134573

**Published:** 2015-07-31

**Authors:** Valentina Nardone, Luca Cistrone, Ivy Di Salvo, Alessandro Ariano, Antonello Migliozzi, Claudia Allegrini, Leonardo Ancillotto, Antonio Fulco, Danilo Russo

**Affiliations:** 1 Wildlife Research Unit, Laboratorio di Ecologia Applicata, Dipartimento di Agraria, Università degli Studi di Napoli Federico II, Portici, Napoli, Italy; 2 Forestry and Conservation, Cassino, Italy; 3 Laboratorio di Ecologia Applicata, Dipartimento di Agraria, Università degli Studi di Napoli Federico II, Portici, Napoli, Italy; 4 Dipartimento di Biologia e Biotecnologie “Charles Darwin”, Università degli Studi di Roma 'La Sapienza', Roma, Italy; 5 School of Biological Sciences, University of Bristol, Bristol, United Kingdom; University of Regina, CANADA

## Abstract

Intra-sexual segregation is a form of social segregation widespread among vertebrates. In the bat *Myotis daubentonii*, males are disproportionately abundant at higher elevations, while females are restricted to lower altitude. Intra-male segregation is also known to occur yet its ecological and behavioural determinants are unclear. We studied male segregation along a river in Central Italy where we tested the following predictions: 1. Upstream ( > 1000 m a.s.l.) males will rely on scarcer prey; 2. To deal with this limitation and exploit a cooler roosting environment, they will employ more prolonged and deeper torpor than downstream (< 900 m a.s.l.) males; 3. Body condition will be better in downstream males as they forage in more productive areas; 4. To cope with less predictable foraging opportunities, upstream males will use more habitat types. Consistent with our predictions, we found that prey were less common at higher altitudes, where bats exhibited prolonged and deeper torpor. Body condition was better in downstream males than in upstream males but not in all summer months. This result reflected a decrease in downstream males’ body condition over the season, perhaps due to the energy costs of reduced opportunities to use torpor and/or intraspecific competition. Downstream males mainly foraged over selected riparian vegetation whereas upstream males used a greater variety of habitats. One controversial issue is whether upstream males are excluded from lower elevations by resident bats. We tested this by translocating 10 upstream males to a downstream roost: eight returned to the high elevation site in 1-2 nights, two persisted at low altitude but did not roost with resident bats. These results are consistent with the idea of segregation due to competition. Living at high altitude allows for more effective heterothermy and may thus be not detrimental for survival, but by staying at lower altitude males increase proximity to females and potentially benefit from summer mating opportunities.

## Introduction

Several forces can drive social segregation, i.e. the tendency to form separate social groups [1] in vertebrates. Sexual segregation is a common form of social segregation and in vertebrates may be often explained in terms of sex-specific habitat requirements and/or sociality. Differences in energy demands, body size, social behaviour, antipredatory needs and breeding phenology are all closely associated with spatial, social and habitat selection differences between sexes [2,3,4]. The two sexes may either segregate spatially or temporally [5]; segregation has been advocated at least in certain cases as a mechanism to mitigate intersexual competition, yet this might only be an effect, rather than the driver, of the phenomenon.

Besides sexual segregation, another (subtler) form of social segregation recorded in vertebrates is intrasexual segregation [6]. The two social patterns may be related for several reasons. For instance, the presence or absence of one sex in social groups may influence within-sex behavioural rates of aggression in the other, leading to different degrees of intrasexual segregation [7], or some males may associate with females as a form of antipredatory mimicry [8] or to increase reproductive success [9]. A common explanation for intrasexual segregation is sex-specific aggressiveness, so that males will only displace males from their vital space, females only females [10,11], but this is unlikely to apply to all known cases.

Sexual segregation in bats is often attributed to different microclimate requirements [12] or prey availability [13]. Many temperate bats exhibit sex-biased segregation with females occurring at lower altitudes than males during the activity season [14]. This spatial segregation is explained in terms of different energetic requirements of the two sexes and often expressed by a biased sex ratio over elevational gradients, with males being more frequent at higher altitudes [15,16]. Adult males—as well as non-breeding females and juveniles of both sexes—may find it advantageous to stay at colder sites (i.e. those at higher altitudes) to save fat reserves more efficiently by torpor [15,16] in the day. In the breeding season, females need to maintain homeothermy for foetal development and the increased energy demands posed by this condition or by subsequent lactation lead them to congregate at lower altitude, where warmer roosts and more productive foraging habitats are found [9,17,18].

The Daubenton’s bat *Myotis daubentonii* (Vespertilionidae. Kuhl, 1817) is a medium-sized vespertilionid strictly associated with aquatic habitats, where prey are either caught on the wing or “trawled” from the water surface by using feet and/or the wing membrane [19]. This species mainly preys upon small dipterans in the Chironomidae family, which constitute the bulk of its diet [20,21]. *M*. *daubentonii* represents an interesting model species to investigate social segregation both between and within sexes: in several regions of Europe adult males are disproportionately abundant at higher elevations, while females are restricted to lower altitudes [16,22,23]. The actual elevational threshold above which only males are found depends on latitude [16,24]. Low-altitude males share summer roosts with females and have been found to take advantage of this proximity by mating in summer besides autumn [9,25] thus achieving a higher reproductive success than high-altitude males [13]. From an energetic point of view, low-altitude males should be able to exploit more productive foraging habitats as insects are known to be more abundant in warmer sites [26] and also gain access to warmer roosting sites. It is unclear why only some males share habitats with females, yet a plausible hypothesis is territoriality, i.e. low-altitude bats (males and/or females) actively exclude some males restricting them to higher elevation in less productive environments [9,16,27].

The energetic costs of living at different elevations may be largely influenced by the frequency and depth of daytime torpor [28,29,30]. In summer, when prey are scarce and/or temperatures are low, *M*. *daubentonii* males may use daily torpor [29,31,32,33] but clearly the energetic significance of torpor will depend on roost temperature (in turn, an effect of altitude-dependent temperature) as well as on how much energy is gained by foraging [33,34].

Although much work has been done on inter- and intrasexual segregation in *M*. *daubentonii* [9,13,16,35], the ecology of male segregation along an altitudinal gradient has yet to be fully assessed. We addressed this by evaluating the effects of altitude on thermal and foraging ecology of male *M*. *daubentonii* and compared insect abundance, body condition, thermoregulation strategies, use of space and habitat selection between two altitude zones along a river. Specifically, we tested the following predictions:

1. Foraging areas located downstream will have higher prey availability so foraging there will be more profitable;
2. To cope with less favourable foraging opportunities, upstream males will rely on torpor to minimize energetic expenditures more than downstream males;
3. If prediction 1, is true, downstream males will be in a better body condition because they feed in more productive areas;
4. To cope with a less productive and predictable environment and track potentially ephemeral food concentrations, upstream males will be more flexible in habitat selection, using a wider variety of habitats;
5. One controversial issue is whether upstream males are excluded from lower elevations by intraspecific competition with resident bats [9,16]. To test this, we translocated upstream males to a low altitude site. We predict that if the competition hypothesis is false, then translocated upstream males will remain in the new area exhibiting no substantial behavioural difference from typical downstream males.

## Materials and Methods

### Field permit

Bat capture, handling and tagging were carried out under licence from the Italian Ministry for the Environment and the Protection of Land and Sea (permit nr 0011284) and the Abruzzo Lazio and Molise National Park (permit nr 0004573/2012)

### Study area

**Fig 1:**
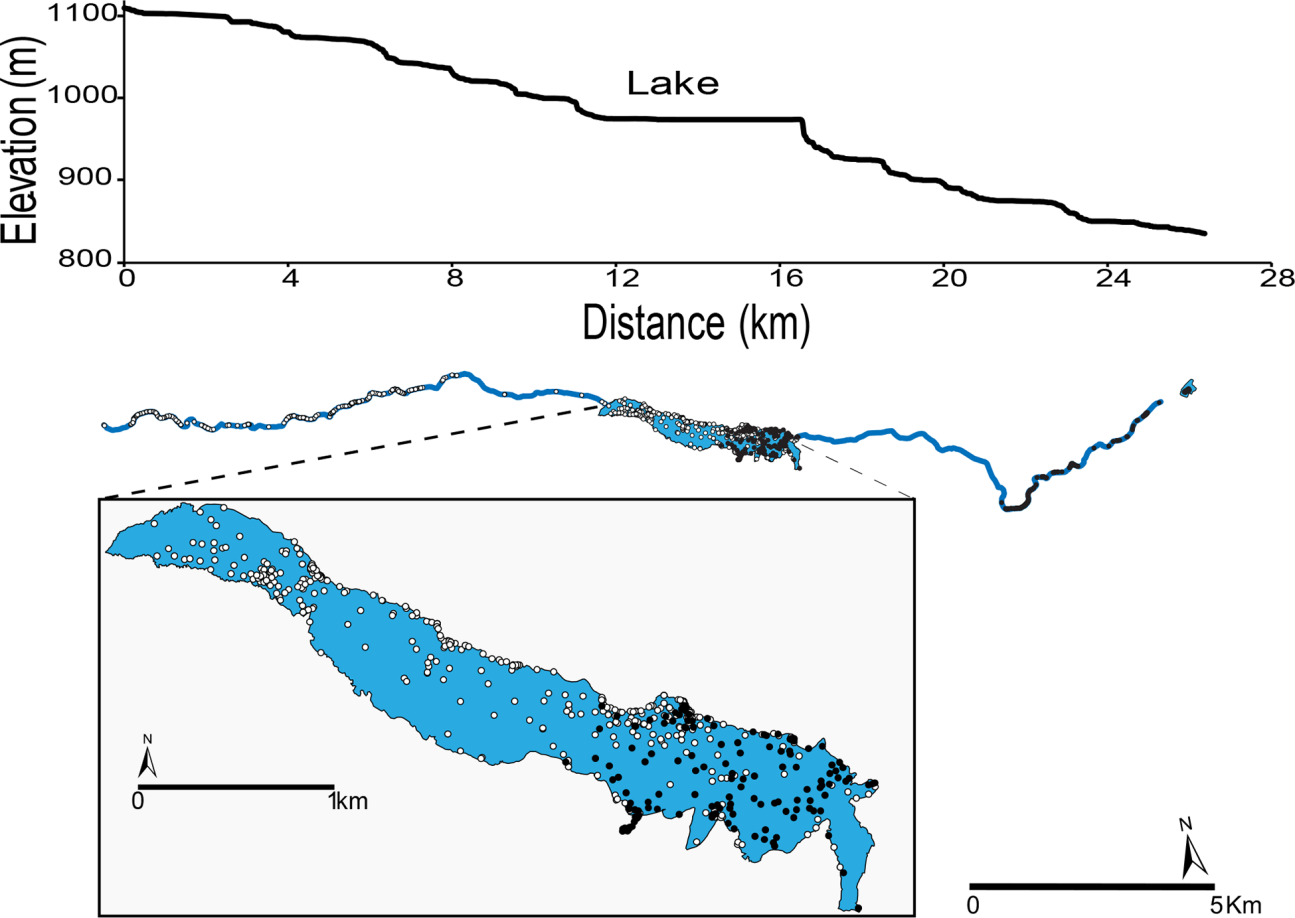
Elevation gradient (above) and map (below) of study area, Sangro river and Barrea Lake (in the Abruzzo Lazio and Molise National Park and its buffer zone, Central Italy) with locations (fixes) of 23 *Myotis daubentonii* detected by radio tracking. Note the overlap of foraging areas used by males (open circles = upstream males, closed circles = downstream males) from the two altitudinal zones in the lake area.

The *M*. *daubentonii* population we investigated was found along a 28-km stretch of the Sangro River (Fig 1), in the Abruzzo, Lazio and Molise National Park and its buffer zone, in Central Italy (41° 45′ 46.8″ N, 13° 58′ 8.4″ E). In the study area the river stretches along an altitudinal gradient of 300 m between 1100 m and 800 m a.s.l. and is mostly characterized by laminar flow with limited turbulence. At ca. 950 m a.s.l the river is blocked by a dam used to produce hydroelectric power, forming an artificial lake (Barrea Lake). Riparian vegetation is well developed along much of the river course and is dominated by *Salix* spp.

Female *M*. *daubentonii* do not occur > 900 m a.s.l. and males at lower altitudes are far less numerous than at higher elevations [16]. Hereafter we refer to “high” and “low” elevation (or “upstream” and “downstream”) as to the altitudinal zones respectively above 1000 m a.s.l. and below 900 m a.s.l.; the Barrea Lake is located at ca. 950 m a.s.l. (Fig 1).

### Prey abundance

During summer 2013 we sampled nocturnal aerial insects using sticky traps [33,36]. Sampling occurred on the same days to control for temporal variation in prey availability across habitats. Based on radiotracking data (see below) we selected three main areas for each of the high and low altitudinal zones as well as at the lake. For each of them, on the river, we chose three trapping sites, at least 50 m apart. At each site we installed two sticky traps, one for each bank, while for each of the three areas on the lake we placed six traps along the bank at least 50 m from each other. The traps were installed 10 cm from the water surface. They consisted of a circular panel (22 cm of diameter) sprayed with glue (Vebi Colla Spray, Vebi Istituto Biochimico s.r.l., Italy). To sample nocturnal insects only, sticky traps were set up at sunset and removed at sunrise over three days. We identified insect families with a stereo-microscope using an identification key [37] and reference material.

### Daytime thermoregulation

We used temperature telemetry [33,38,39] to investigate the thermoregulation strategies of *M*. *daubentonii* adult males in the two altitude zones. During July and August of 2012 and 2013 we mistnetted bats over the river in the surroundings of two known roosts (bridges stretching across the river) respectively at high and low elevation. In both roosts ca. 100–150 bats roosted together. At high elevation only males were present in the roost, while downstream both sexes occurred in the colony. At the time of the study male testes were either inconspicuous or moderately evident but no individual had filled epididymides. For each individual we assessed age and sex [40,41]. Bats were fitted with temperature-sensitive radio-transmitters (LB-2NT, Holohil Systems, Ontario, Canada) using Skinbond^(R)^ surgical cement. The combined mass of the transmitter (0.36 g) and glue did not exceed 5% of the bat’s body mass. Pulse emission rates of the transmitters changed as a function of skin temperature (T_s_) which was inferred using unit-specific calibration curves provided by the manufacturer [33,38,39]. Transmitter signals were detected using an R-1000 telemetry receiver (Communications Specialists, Inc., CA) connected with a Yagi antenna. In daytime (meant as the time between a bat’s return to roost and its subsequent emergence) we timed 21 pulses 3 times every 15 minutes for all bats [33]. We also measured ambient temperature (T_a_) with a digital thermometer (±0.1°C) placed in the shade near the roost at a height of 1.5 m. Roost structure precluded us from measuring roost’s internal temperature.

Willis and Brigham [42] showed that T_s_ and body core temperature are strongly correlated and do not differ > 6°C. We categorized as torpid bats those whose T_s_ was < 6°C relative to the temperature measured 15 minutes before emergence from roost [32]; the latter was assumed to be typical of an active, fully homeothermic bat. We calculated the heterothermy index (HI) [43], which expresses the temporal variation in skin temperatures in a certain sampling period in relation to the active optimal skin temperature recorded 15 minutes before roost emergence [32]. Higher HI values express a greater magnitude of heterothermy. Sunrise and sunset times changed > one hour during the sampling period. To make sure our HI referred to bats in the roost we restricted its calculation to the time comprised between 6.00 AM and 8:00 PM when all bats were day-roosting. We obtained data from twenty-two adult males (*n* upstream = 12; *n* downstream = 10).

### Body condition

We assessed body condition using forearm length (FAL, mm) and body mass (g) of 153 *M*. *daubentonii* adult males mistnetted in 2000–2013 within the boundaries of the study area along the Sangro river’s altitudinal gradient. FAL and body mass were measured respectively with a caliper to the nearest 0.1 mm and a digital scale to the nearest 0.1 g. For each bat we calculated the Scaled Mass Index (SMI) [44]. We chose to use SMI because for both small terrestrial mammals [44] and bats [45] it is regarded as a robust indicator of the body condition which best accounts for variation linked with size, age and sex [46].

### Use of space and night activity

We radiotracked 23 adult males mistnetted in July-August 2012–2013 and fitted them with temperature-sensitive radio-transmitters (model LB-2NT, Holohil Systems, Ontario, Canada) from dusk emergence to their return to the roost (Table 1). Bat locations (hereafter termed fixes) were obtained by cross-bearing and, where applicable, “homing-in” [47]. We assessed the degree of accuracy by locating stationary active tags in several sites across the study area and comparing their actual position with that estimated by radiotracking. In all cases the error was negligible (< 5 m). Spatial analysis was carried out with ArcView 3.1 (ESRI). All fixes were mapped using the “Radiating Line” ArcView Extension (Jenness Enterprises, http://www.jennessent.com/arcview/radiating_lines.htm).

**Table 1:** Date of capture, altitude of capture and roosting, biometry (FAL = forearm length, BM = body mass) and tracking details of 29 male *Myotis daubentonii* radiotracked at the Abruzzo, Lazio and Molise National Park and its buffer zone, in Central Italy.

Bat code	Date of capture	Altitude	FAL (mm)	M (g)	Days tracked	*N* fixes
170433	04/07/2012	High	37.3	7.1	7	66
170438	04/07/2012	High	35.4	7.1	3	66
170443	04/07/2012	High	36.4	6.9	9	56
170439	07/07/2012	Low	35.9	7.1	7	53
170449	07/07/2012	Low	38.4	7.1	7	60
170448	15/07/2012	High	36.2	7.1	3	89
170440	20/08/2012	Low	37.5	6.1	7	59
170446	20/08/2012	Low	36.7	6.5	7	69
174701	20/08/2012	Low	37	6.9	7	91
174703	25/08/2012	High	37.3	6.8	4	79
174706	25/08/2012	High	35.9	6	6	73
174710	25/08/2012	High	36.8	6.5	5	64
180021	01/07/2013	Low	36.9	7.8	3	81
180023	01/07/2013	Low	36.5	6.5	3	64
180030	01/07/2013	Low	34.2	6.3	4	80
180040	01/07/2013	Low	37.3	6.9	3	77
180032	09/07/2013	High	37.1	5.3	6	69
180027	10/07/2013	High	37.8	7.1	4	66
180038	10/07/2013	High	37.2	7.7	4	60
180022	22/08/2013	High*	38.4	6.1	5	62
180025	22/08/2013	High*	36.3	6.3	2	0**
180026	22/08/2013	High*	36.7	6.7	8	0**
180028	22/08/2013	High*	38.6	7	3	69
180029	22/08/2013	High*	38	7.2	2	80
180031	22/08/2013	High*	36.7	7.7	8	56
180034	22/08/2013	High*	35.5	6.5	4	5
180035	22/08/2013	High*	36.5	6.5	5	85
180037	22/08/2013	High*	37	6.5	6	57
180039	22/08/2013	High*	36.3	6.2	3	7
Mean ± SD			37.3 ± 0.6	6.5 ± 0.7	5 ± 2	64.6 ± 19.8

*N* fixes = number of foraging locations recorded for each bat. Bats labelled with codes 180022, -28, -29 and -37 were also used for spatial or thermal analyses after returning to high altitude.

* = Bats that were translocated to low elevation.

** = No foraging fixes recorded, but bat presence ascertained on the basis of commuting/roosting fixes.

A 2380 km^2^ small-scale vegetation map was generated *a posteriori* by carrying out photo-interpretation of the riparian vegetation within a 10-m spatial buffer from the banks of all water bodies. Photo interpretation relied on 0.2 m/px digital ortophotos—i.e. aerial photographs corrected so that the scale is uniform—at a nominal scale of 1: 5.000. We classified the habitats potentially relevant for *M*. *daubentonii* as follows: river with riparian vegetation on both banks (17%), river with riparian vegetation on one bank (2%), river with no riparian vegetation (2%), lake shore with riparian vegetation (7%), lake shore with no riparian vegetation (2%), lake-interiors (64%) and flooded *Salix* spp. woodland (6%; S1, S2 and S3 Figs).

To assess habitat selection we considered only foraging fixes and included bats for which ≥ 50 fixes were obtained. The number of fixes falling in each habitat category was defined by carrying out a GIS spatial join operation between the shape files containing bat fixes and habitat types. Our radiotracking data clearly showed that when foraging or commuting, bats never left the main river or other minor water bodies in its immediate surroundings: we thus refrained from using Minimum Convex Polygons for habitat selection analysis as these would have included large proportions of unused habitat. Instead, we concentrated on the habitat actually suitable to bats comprised within the spatial buffer used for photointerpretation.

To calculate the maximum distance travelled on a night from the roost, we considered: 1) the straight distance between the roost and the farthest location reached on a given night; and 2) the length of the actual route covered by bats along the waterways they followed.

Finally, we compared the time spent night-roosting between upstream and downstream males, calculated as the total amount of time a bat spent inactive (not flying) from emergence to sunrise.

### Translocation experiment

In August 2013, we mistnetted 10 *M*. *daubentonii* adult males at a high altitude roost, fitted them with temperature-sensitive radio-transmitters (model LB-2NT, Holohil Systems, Ontario, Canada) and promptly transferred them to a downstream roost hosting over 100 adult bats of both sexes. Bats were manually introduced in the downstream roost within 3 hrs to capture. Over the subsequent 12 days we monitored their nightly movements and daytime skin temperature. Bats that returned to high altitude were included in the analysis as part of the upstream male sample, but data for spatial and thermal analyses were collected respectively only after at least two and eight days had elapsed since their return. After this time their behaviour was identical to that of radio-tagged high altitude bats that had not been translocated.

### Statistical analysis

We applied General Linear Models (GLM ANOVA) followed by Tukey’s post-hoc tests to compare the number of chironomids (Diptera Chironomidae) caught along the altitudinal gradient. We focused on such insects because they represent *M*. *daubentonii*’s staple food, e.g. [20,21], and can thus be used to assess the insect abundance of foraging areas.

The relationship between HI and T_a_ was explored with Pearson correlation tests_;_ HI was also compared between upstream and downstream males using a GLM ANOVA. The same test followed by Tukey’s post-hoc comparisons was also used to explore the effects of altitude and month of capture (June, July and August) on SMI. To assess habitat selection we performed a compositional analysis [48] in which the percent foraging fixes recorded for each habitat represented the “used” portion whereas the percent habitat occurrence expressed habitat availability.

We compared the mean and maximum distances travelled on a night and night-roosting time between upstream and downstream males by GLM ANOVA. All analyses except compositional analysis were performed with Minitab 13.1 (State College, PA: Minitab, Inc.). Compositional analysis was performed with R’s “*adehabitat*” package (R Core Development Team) [49]. Statistical significance was assumed when P < 0.05.

## Results

### Prey abundance

We collected a total of 3111 chironomids. The mean number of chironomids caught differed significantly among the three study area sectors, and was highest at the lake, intermediate downstream and lowest upstream (lake 222 ± 137, downstream 80 ± 33, upstream 44 ± 23; GLM ANOVA, F_2,24_ = 22.95, P < 0.005 and Tukey’s post-hoc tests).

### Daytime thermoregulation

**Fig 2:**
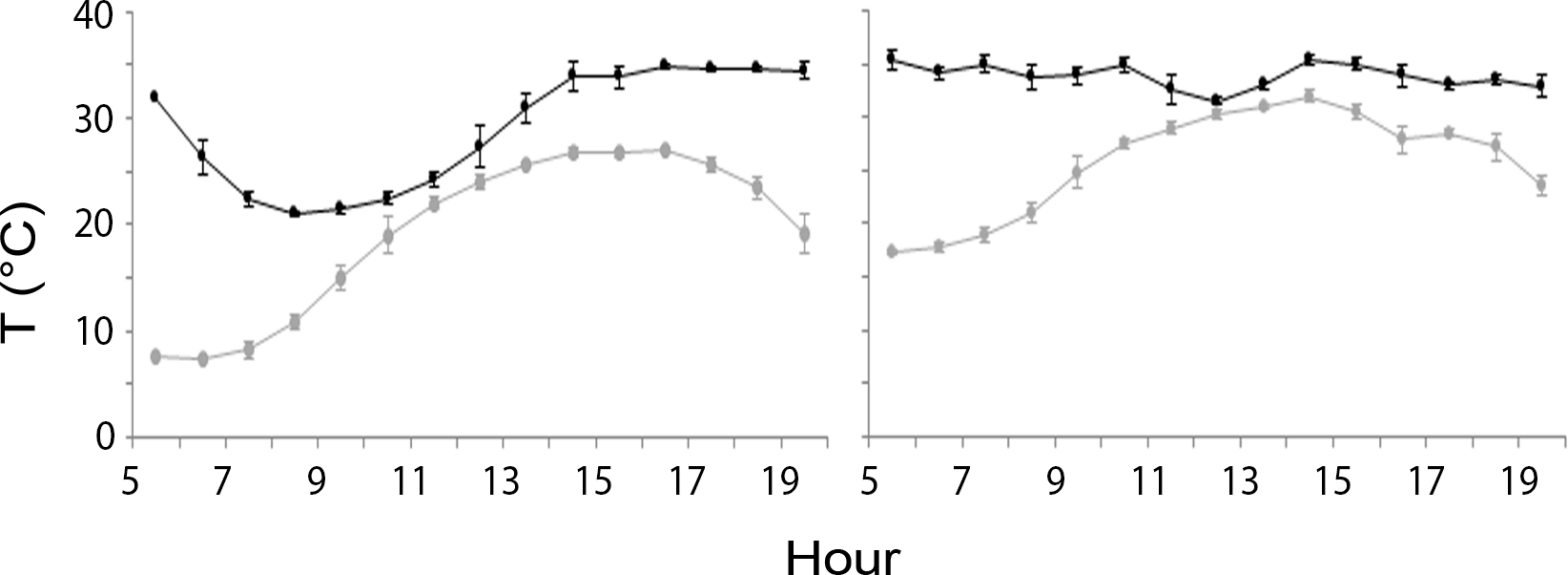
Daytime skin temperature patterns respectively representative of an upstream (left) and a downstream male (right) *Myotis daubentonii* simultaneously recorded in August 2012 at the Abruzzo Lazio and Molise National Park and its buffer zone (Central Italy). Skin temperature is given by the black line and ambient temperature by the grey line. When upstream males entered the roost, a body temperature drop was typically observed, leading to heterothermy, while downstream males remained mostly homeothermic in daytime.

Skin temperature patterns and thermal behaviour clearly differed between elevational zones. Typically, when upstream males returned to the roost, we recorded skin temperature to drop and bats became heterothermic, whereas downstream males remained mostly homeothermic in daytime (Fig 2). On average the maximum T_s_ of upstream males was 35.7 ± 1.8°C (mean ± standard deviation), the minimum T_s_ was 22.7 ±2.4°C and the daily drop in T_s_ was 13 ± 3.5°C. The maximum and minimum T_s_ of downstream males were respectively 36.4 ± 0.8°C and 28.1± 2.6°C, with a daily T_s_ drop of 8.3 ± 2.9°C. The Heterothermy Index (HI) was significantly higher (7.61 ± 3.00) for upstream males than for those downstream (4.39 ± 2.92) (GLM ANOVA, F_1,20_ = 6.39, P < 0.05). HI was also negatively correlated with T_a_ (r = -0.57; P < 0.005).

### Body condition

Scaled Mass Index of 153 males captured in 2000–2013 was significantly higher for downstream males than for those upstream (GLM ANOVA, F_1,149_ = 14.56, P < 0.005); month of capture showed a significant effect on SMI only in June (F_1,149_ = 4.90, P < 0.01), reflecting the fact that later in the summer body condition dropped in downstream males so that by July its value approached that of upstream males.

### Use of space and night activity

Bats were only recorded over water or near riparian vegetation (Fig 1). Except for translocated individuals, upstream males never switched to downstream roosts and vice versa. For upstream males, compositional analysis led to the following ranking (where significant differences occur, habitats are separated with >>>): river with riparian vegetation on both banks > lake shore without riparian vegetation > lake shore with riparian vegetation > lake interiors > river with riparian vegetation on one bank > flooded *Salix* spp. woodland >>> river without riparian vegetation on banks. For downstream males we found: river with riparian vegetation on both banks >>> lake shore with riparian vegetation > lake shore without riparian vegetation > river without riparian vegetation on banks > river with riparian vegetation on one bank > lake-interiors > flooded *Salix* spp. woodland. Noticeably, foraging areas of upstream and downstream males over the lake overlapped (Fig 1). Upstream males flew longer nightly distances than downstream males (GLM ANOVA, Table 2) and night-roosted for a longer time than the latter (GLM ANOVA, F_1,11_ = 7.42, P < 0.05; Table 2). Although some downstream males moved upstream and reached the lake to forage, upstream males never moved to the downstream area. Three of the upstream males switched roosts every day while the remaining were loyal to the same site; downstream males shared the roost with females and never switched.

**Table 2:** Distances travelled, night-roosting time and statistical comparisons for 14 upstream and 9 downstream *Myotis daubentonii* males radiotracked at the Abruzzo, Lazio and Molise National Park and its buffer zone, in Central Italy.

	Actual route length covered (km)	Straight distance (km)	Max actual route length covered (km)	Max straight distance (km)	Inactivity time (% night)
Upstream	7.3 ± 2.9	6.5 ± 2.8	10.3 ± 4.1	9.2 ± 3.8	33 ± 12
Downstream	2.4 ± 2.1	2.1 ± 1.8	3.6 ± 2.9	3.1 ± 2.5	18 ± 7
GLM, ANOVA	F_1,21_ = 19.12	F_1,21_ = 17.84	F_1,21_ = 17.86	F_1,21_ = 18.04	F_1,11_ = 7.42
	P < 0.005	P < 0.005	P < 0.005	P < 0.005	P < 0.05

We considered both the straight distance from the roost to the farthest location reached on a given night and the length of the actual route covered by bats following the waterways; inactivity time is the total amount of time a bat spent inactive from emergence to sunrise.

### Testing the competition hypothesis

Eight of ten upstream males we translocated downstream returned to high altitude areas in one or two nights, two stayed at low elevation for longer. One male stayed two days at the roost to where it had been translocated, then moved ca. 5.5 km farther downstream where it spent at least another 8 days (data collection ended due to battery failure). The other bat roosted with downstream males and females only on the day following translocation, then moved to a nearby bridge where it apparently roosted alone for four days before moving back. This bat only showed brief foraging bouts in a foraging area where resident conspecifics hunted but spent most of the time night-roosting. Both subjects foraged much less than the other bats, as shown by the fact that they night-roosted longer (mean percent time of night spent roosting ± SD, 60.1 ± 22.6) than upstream (32.7 ± 12.2) and downstream (18.1 ± 7.3) males (GLM ANOVA, F_2,12_ = 11.94, P < 0.005).

## Discussion

Our study offers a comprehensive picture of thermal and foraging ecology of male *M*. *daubentonii* living upstream and downstream of a lake, along an elevational gradient that despite its limited slope (ca. 300 m) led bats to adopt completely different strategies. Our altitude gradient is greater that those considered in UK studies, where intrasexual segregation of males in *M*. *daubentonii* was also analysed [9,13]. One study [9] recorded spatial use differences between upstream and downstream males along a ca. 100 m gradient, whereas a more recent analysis [13] was extended farther downstream, categorizing bats according to three elevational zones (upper-elevation site > 200 m a.s.l.; mid-elevation site 100–200 m a.s.l.; low-elevation site < 100 m a.s.l.). We assume that our “upstream” and “downstream” males correspond to the extremes of the altitude gradient considered by the latter study [13]. The situation we studied also differed from that of Angell et al. [13] because in our study area the artificial lake physically separated the upstream and downstream river stretches, creating an additional habitat type.

Consistent with our first prediction, we found that downstream areas are more productive, so bats roosting there potentially have an energetic advantage. These bats moved less to reach profitable feeding sites and covered shorter distances between foraging sites [9]. This sets the scene for the hypothesis that females and/or dominant males would select more productive foraging areas at low altitudes excluding subordinate males at higher elevations [9,15,16,27].

By sampling chironomids, we confirmed what as indirectly assessed by Angell et al. [13] by counting feeding buzzes—sequences of echolocation pulses broadcast when attempting to catch prey [50].

Chironomids are found in a range of habitats and water conditions; however, many species in this genus are tolerant of organic pollution and may be favoured by eutrophication [51,52]. In addition, in their larval stages they are major components of benthos in backwater, therefore abound in lakes where they may colonize both microphytes and soft sediment [53], which explains why they exhibited peak abundance at the lake as well as downstream, where human settlements are more widespread and river water more subjected to organic input. Chironomids also concentrate where air and water temperatures are higher [54] so they are more likely to occur downstream, favouring foraging activity there. Only a previous study [9] besides ours radiotracked upstream males and downstream males and unlike in our case found no foraging area overlap, whereas in our case downstream males moved upstream to forage at the lake on some nights which was also used by upstream males. Our results may differ because of the presence of the lake connecting the areas upstream and downstream—an especially profitable foraging habitat for bats from both elevational zones. It is also important to note that although downstream males in several cases moved upstream (to the lake) to forage, upstream males never moved downstream of the lake. Whether foraging sites downstream of the lake (however productive) were too distant to represent convenient destinations for upstream males, or the latter’s access to those areas was precluded by resident bats has yet to be ascertained. Noticeably, downstream males flew past the dam to reach the lake, so the dam was not a barrier [55].

Upstream males exhibited more prolonged and deeper torpor than did those at low altitudes, in agreement with our second prediction. Our results confirmed what was found by Encarnação et al. [56] that related individual variation in torpor expression to habitat characteristics. Besides hibernating in winter, bats from temperate areas use daily torpor to minimize energy loss year round [57], yet torpor’s depth and duration depend on environmental conditions [39]. We could not measure roost temperature because the actual roosting spaces were not accessible. We assume that roost’s internal temperatures reflected those we measured outside. The colder ambient temperatures found upstream allow males to employ heterothermy and thus save energy. Downstream males were probably unable to do so because of the warmer roost temperatures, which would partly explain why they remained homeothermic in daytime. Our results agree with those of Becker et al. [33] which related depth and duration of torpor in male *M*. *daubentonii* with altitude.

The negative relationship we found between the heterothermy index and ambient temperature is also consistent with the hypothesis that thermoregulation strategies in male *M*. *daubentonii* are mainly influenced by T_a_ [39,58]. As in previous studies [9,16] males caught downstream had a better body condition yet we recorded a drop of the latter which by the end of summer tended to equal that of upstream males. Assuming downstream and upstream males are loyal to the same areas year round, we suggest that the better body condition of the former early in the season is due to the higher insect abundance of the feeding sites they may exploit soon after hibernation, or to the milder winter temperatures they face, allowing more frequent arousals from hibernation to forage [59,60,61]. Fewer opportunities to use torpor in summer, the onset of spermatogenesis and mating activity at that time [25,9] or perhaps stronger competition at feeding sites with females and volant juveniles may all potentially explain why body condition of downstream males dropped during the season. In male colonies of *M*. *daubentonii* in Germany a pattern with body mass increasing over the year was reported [62].

The maximum distance travelled during the night by radiotracked bats are longer than those reported by Senior et al. [9] in their study of intra-male segregation of *M*. *daubentonii*, although the greatest distances travelled overnight from upstream males are similar to those we recorded. Unlike in that case [9], our upstream males were less active during the night than downstream ones.

Upstream males returned to roosts earlier than downstream males and quickly became torpid. This is likely to be a strategy to preserve energy in a cool roosting environment when insect abundance is too low to make foraging beneficial.

As previously reported [63,64], downstream *M*. *daubentonii* preferred to forage where riparian vegetation is available. The latter shelters foraging spots from wind, keeping the water surface calm—which helps echolocation in trawling bats: [63,65,66]–and allowing insects to congregate in swarms [67]. Besides, riparian vegetation is also an important habitat for insect reproduction [67]. This behaviour is also known for another trawling species, *Myotis capaccinii* ([68]–but see [69], [70]).

Downstream males showed a strict selection pattern as they mainly hunted at river spots with abundant riparian vegetation whereas upstream males were more generalist, simply preferring river areas without vegetation on banks and making a large use of all other habitats. By selecting a broader range of habitats and covering longer distances to find suitable foraging areas [9], upstream males may gain access to temporarily available food concentrations—an important strategy in a less productive and unpredictable foraging environment such as that found at higher altitudes.

Our translocation experiment was consistent with the competition hypothesis as upstream males mostly homed back to their upstream quarters after being translocated to low altitude except two, which stayed where they had been moved but got access to sub-optimal roosting and foraging resources.

*M*. *daubentonii* is territorial at least in foraging sites, where chases are commonly observed [71]. Social groups of *M*. *daubentonii* are centred on females, who have been hypothesized to be dominant over males, excluding them from territories with higher quality foraging habitats [9,15,16,27]. Inter-sex associations of *M*. *daubentonii* are less frequent than in other bat species and their composition has been found to change across years, but males can be tolerated in the roosting areas of female social groups if they comprise high-quality foraging habitats [72].

In summary, we showed that downstream areas are more productive and that the best foraging sites are closer to downstream roosts, yet this only confers a moderate advantage to male *M*. *daubentonii* dwelling in those areas in terms of body condition, which then tends to decline over the active season and reaches the values recorded in upstream males. The strategies of upstream males, including a greater use of daytime torpor, frequent night-roosting and less selective habitat selection, seem to mostly compensate for the disadvantages linked with foraging in less productive areas. Overall this picture is in agreement with the fact that the main advantage for downstream males is reproductive, since those bats are offered an extra-chance for mating [9,13] besides autumn swarming [9,73,74]. However, it cannot be ruled out that living downstream also confers a survival benefit by allowing males’ access to more productive areas early in the active season where they may quickly replenish their fat stores and thus mitigate mortality following hibernation. Why some males stay upstream, losing out on these benefits, remains unclear.

When translocated to low altitude, upstream males either returned promptly to their original areas or stayed downstream but were confined to suboptimal roosting sites and foraged little, making a larger use of torpor. Whether this is the effect of those males being displaced by resident individuals or a phylopatric response is unknown, but our experiment cannot refute the displacement hypothesis, hopefully encouraging further research. If resident males displace those residing upstream, competition for females could be the driving factor behind male-male aggressiveness [11]; alternatively, males found upstream might be displaced there by females, perhaps based on the detection of fitness attributes that are, to date, unknown. Should this be the case, body condition would not be a suitable proxy for individual fitness as its difference between the two elevational zones is only transient and disappears ahead in summer.

## Supporting Information

S1 Fig Occurrence of habitat types within the study area at high elevation. Green = river/lake shores with riparian vegetation, black = river/lake shores with no riparian vegetation, turquoise green = flooded *Salix* spp. woodland, blue = lake core.
(TIF)

S2 Fig Occurrence of habitat types within the study area for Barrea Lake. Green = lake shores with riparian vegetation, black = lake shores with no riparian vegetation, turquoise green = flooded *Salix* spp. woodland, blue = lake core.
(TIF)

S3 Fig Occurrence of habitat types within the study area at low elevation. Green = river/lake shores with riparian vegetation, black = river/lake shores with no riparian vegetation, turquoise green = flooded *Salix* spp. woodland, blue = lake core.
(TIF)
