# Supplementary figures and images for: How to Be a Male at Different Elevations: Ecology of Intra-Sexual Segregation in the Trawling Bat Myotis daubentonii
Source: PLoS One. 2015 Jul 31;10(7):e0134573. doi: 10.1371/journal.pone.0134573 (PMC4521842; doi:10.1371/journal.pone.0134573)

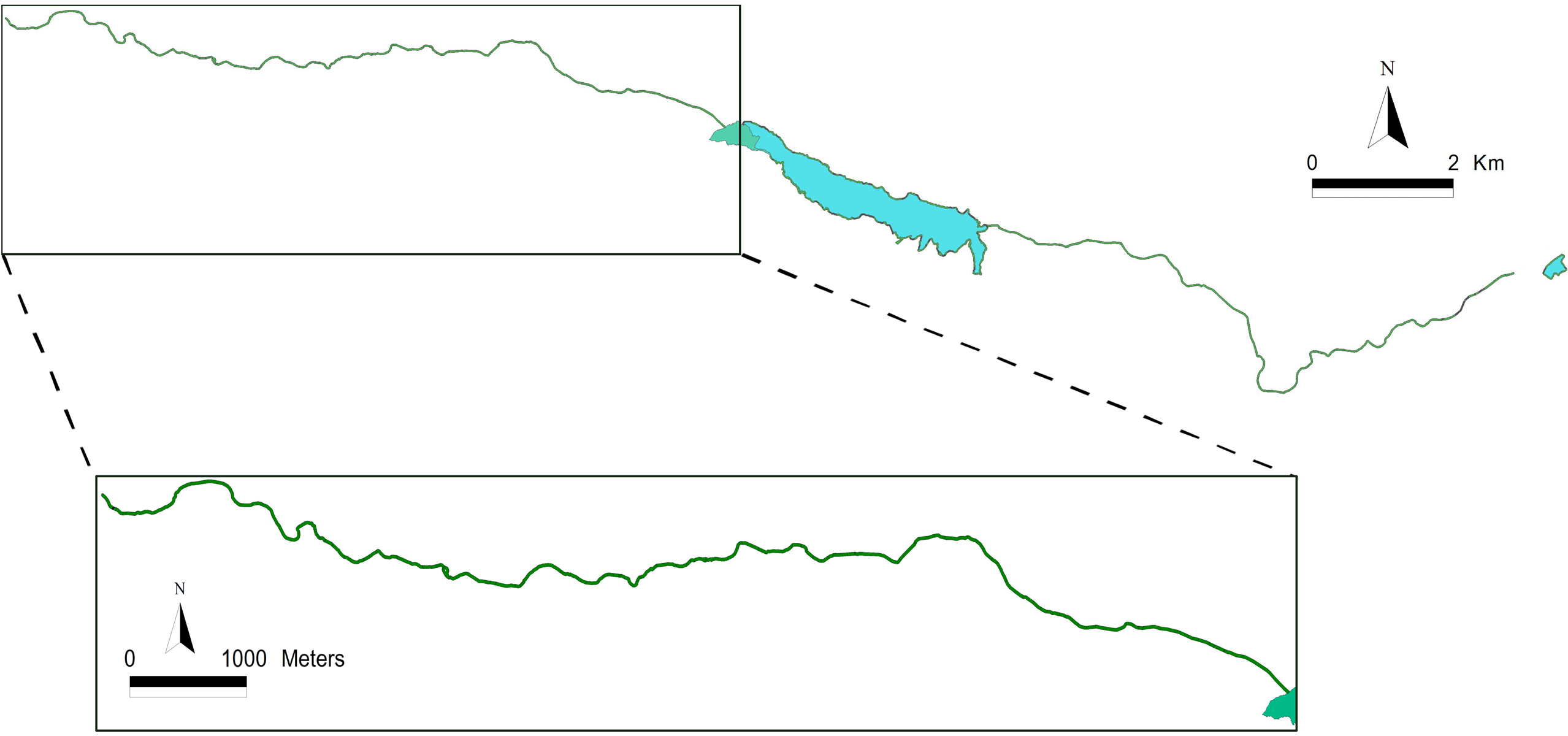

Supplement: S1 Fig — Green = river/lake shores with riparian vegetation, black = river/lake shores with no riparian vegetation, turquoise green = flooded Salix spp. woodland, blue = lake core. (TIF) [file pone.0134573.s001.tif]

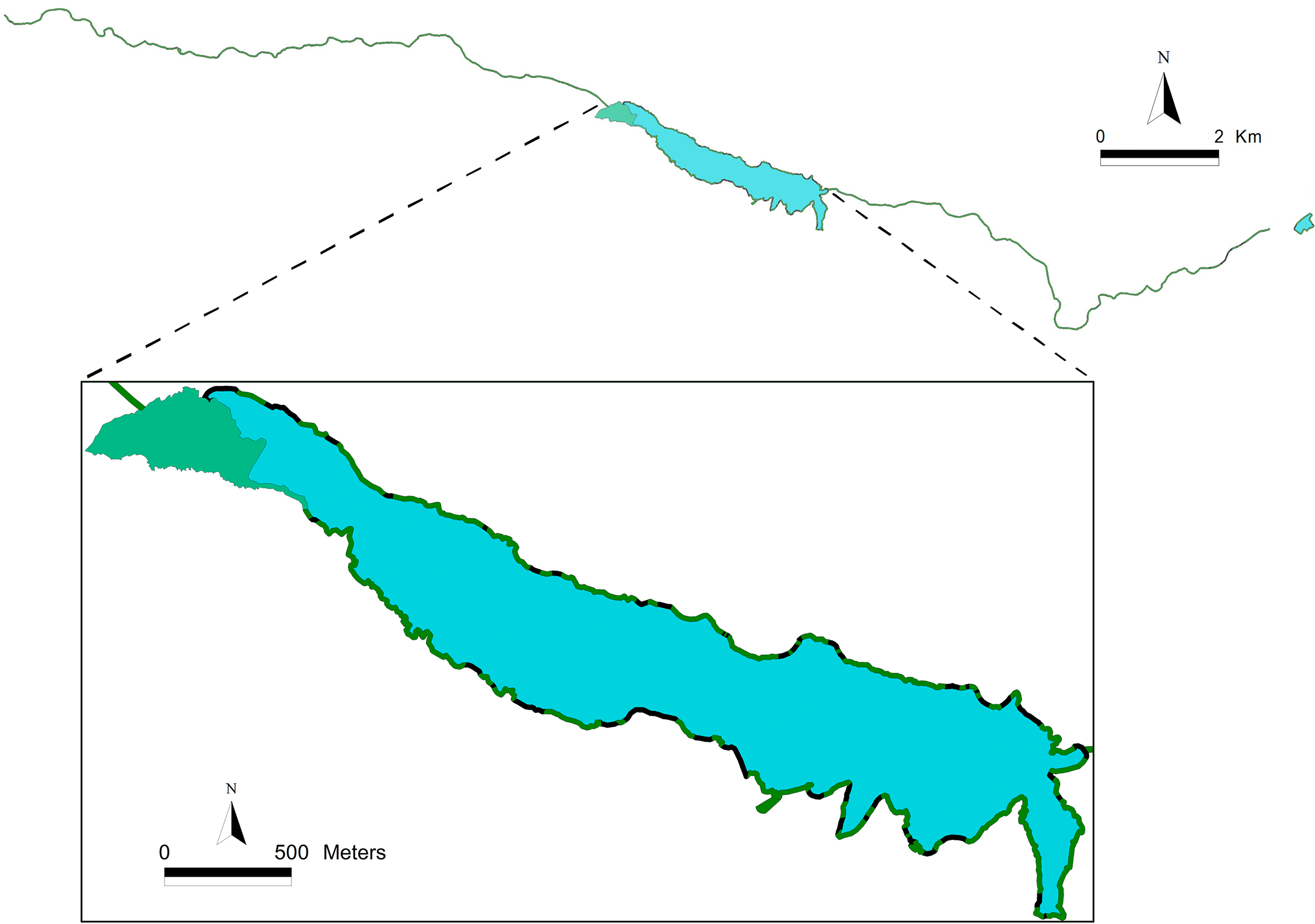

Supplement: S2 Fig — Green = lake shores with riparian vegetation, black = lake shores with no riparian vegetation, turquoise green = flooded Salix spp. woodland, blue = lake core. (TIF) [file pone.0134573.s002.tif]

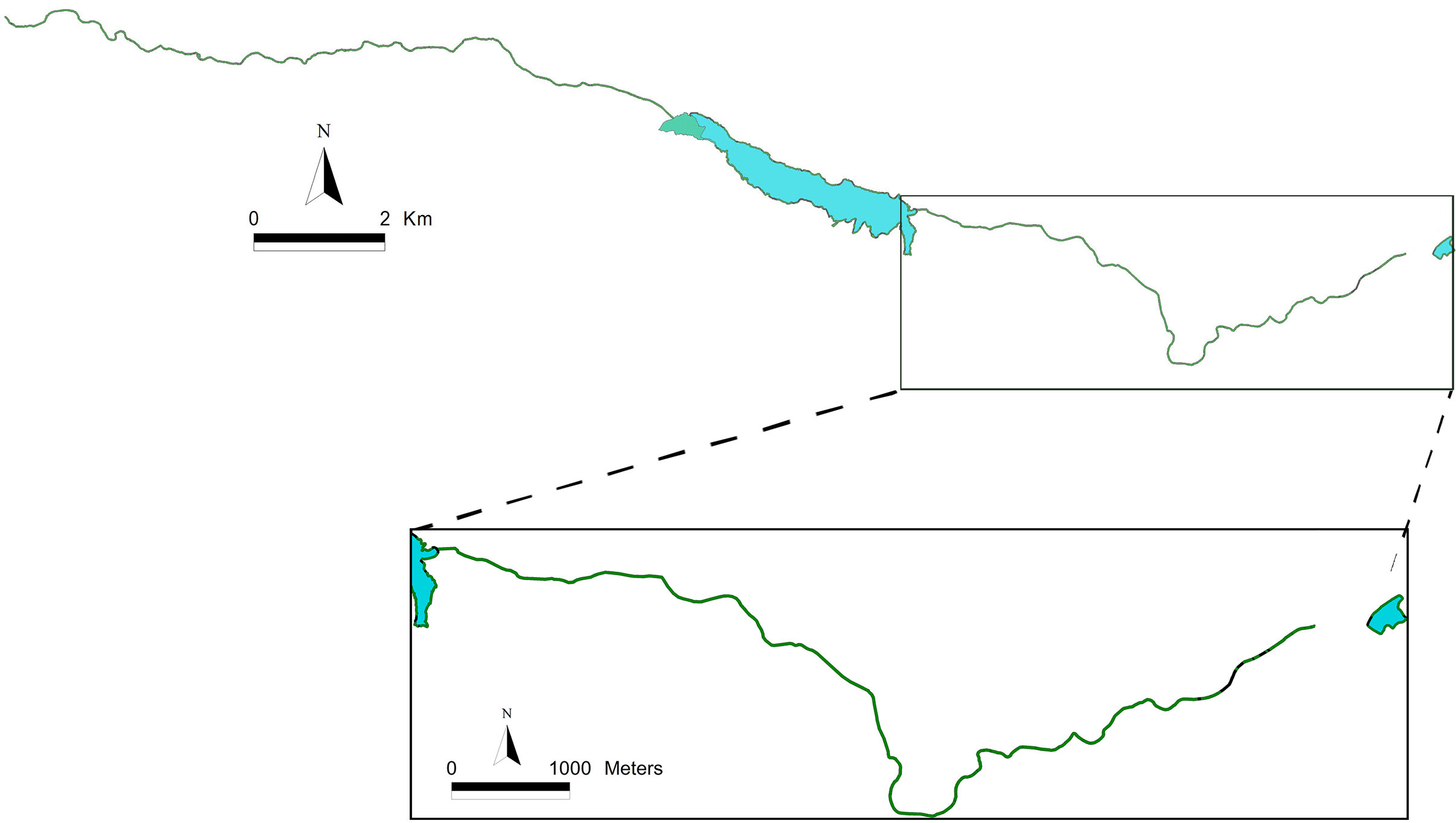

Supplement: S3 Fig — Green = river/lake shores with riparian vegetation, black = river/lake shores with no riparian vegetation, turquoise green = flooded Salix spp. woodland, blue = lake core. (TIF) [file pone.0134573.s003.tif]
